# The Tangibility of Personalized 3D-Printed Feedback May Enhance Youths’ Physical Activity Awareness, Goal Setting, and Motivation: Intervention Study

**DOI:** 10.2196/12067

**Published:** 2019-05-31

**Authors:** Sam Graeme Morgan Crossley, Melitta Anne McNarry, Parisa Eslambolchilar, Zoe Knowles, Kelly Alexandra Mackintosh

**Affiliations:** 1 School of Sport and Exercise Sciences Applied Sports Technology Exercise and Medicine Research Centre Swansea University Swansea United Kingdom; 2 School of Computer Science and Informatics Human Factors Technology Research Priority Area Cardiff University Cardiff United Kingdom; 3 School of Sport and Exercise Sciences Physical Activity Exchange Liverpool John Moores University Liverpool United Kingdom

**Keywords:** behavior change, health education, feedback, self-monitoring, accelerometry, schools, adolescent, child

## Abstract

**Background:**

In the United Kingdom, most youth fail to achieve the government guideline of 60 min of moderate to vigorous physical activity (MVPA) daily. Reasons that are frequently cited for the underachievement of this guideline include (1) a lack of awareness of personal physical activity levels (PALs) and (2) a lack of understanding of what activities and different intensities contribute to daily targets of physical activity (PA). Technological advances have enabled novel ways of representing PA data through personalized tangible three-dimensional (3D) models.

**Objective:**

The purpose of this study was to investigate the efficacy of 3D-printed models to enhance youth awareness and understanding of and motivation to engage in PA.

**Methods:**

A total of 39 primary school children (22 boys; mean age 7.9 [SD 0.3] years) and 58 secondary school adolescents (37 boys; mean age 13.8 [SD 0.3] years) participated in a 7-week fading intervention, whereby participants were given 3D-printed models of their previous week’s objectively assessed PALs at 4 time points. Following the receipt of their 3D model, each participant completed a short semistructured video interview (children, 4.5 [SD 1.2] min; adolescents, 2.2 [SD 0.6] min) to assess their PA awareness, understanding, and motivation. Data were transcribed verbatim and thematically analyzed to enable key emergent themes to be further explored and identified.

**Results:**

Analyses revealed that the 3D models enhanced the youths’ awareness of and ability to recall and self-evaluate their PA behaviors. By the end of the study, the youths, irrespective of age, were able to correctly identify and relate to the government’s PA guideline represented on the models, despite their inability to articulate the government's guideline through time and intensity. Following the fourth 3D model, 72% (71/97) of the youths used the models as a goal-setting strategy, further highlighting such models as a motivational tool to promote PA.

**Conclusions:**

The results suggest that 3D-printed models of PA enhanced the youths’ awareness of their PA levels and provided a motivational tool for goal setting, potentially offering a unique strategy for future PA promotion.

## Introduction

### Background

The government of the United Kingdom recommends that youth (children and adolescents) aged 5 to 18 years should engage in 60 min of moderate-to-vigorous physical activity (MVPA) every day [1] to accrue associated physiological [2,3] and psychosocial health benefits [4,5]. However, only 23% and 20% of boys and girls, respectively, aged 4 to 15 years in the United Kingdom meet these minimum levels of physical activity (PA) [6], with almost 50% of the youths failing to achieve even half the recommended levels [7]. The frequently cited reasons for youth underachievement of the PA guideline are thought to be their lack of awareness of their physical activity levels (PALs) [8-10] and a lack of understanding of what activities and different intensities of PA *count* toward the daily target [11-18]. Given that adults also show a lack of awareness of their PALs [19], have limited knowledge of their respective PA target, and struggle to appropriately identify activity intensities [11], addressing these issues during childhood is important for fostering healthy lifestyle behaviors that can continue into adulthood [20,21].

Based on Weinstein’s [22] Precaution Adoption Process Model (PAPM) from the Stages of Change [23], an individual can only be expected to proceed to the contemplation stage when they become aware that their behaviors are not optimal, such as “I do this much MVPA but this much MVPA is recommended” [11]. In a similar way, the Goal Setting Theory [24] notes that setting specific and challenging, yet achievable, goals, in conjunction with feedback regarding performance toward goal attainment, is important to enhance an individual’s self-efficacy (ie, an individual’s belief to perform a behavior) and health behavior change. In this regard, personalized feedback that represents an individual’s PALs in contrast to the recommended level of activity (ie, acting as a goal) is recognized as an important method for raising one’s awareness of their PA behaviors and subsequent behavior change [25]. Therefore, for health education to be successful in youths, efforts must be made to first raise an awareness and understanding of their PALs in the form of personalized feedback [8] that supports goal attainment (ie, meeting the recommended guideline) [26]. To make personalized feedback effective, it is important that it is visually stimulating and meaningful to the individual [27,28], as *seeing* makes knowledge credible [29], and greater visibility of feedback contributes to be an added responsibility to act [30,31]. Most personalized feedback is presented through digital on-screen displays (eg, mobile phones or activity tracker displays) [32-38]; however, with recent advancements in three-dimensional (3D) printing technology, Khot et al [39] explored an innovative approach to displaying adults’ heart rate data through tangible 3D-printed artifacts to represent a day of PA. This novel approach demonstrated that the visual and tactile nature of the feedback increased adults’ awareness of and reflection on their personal PA [39]. Indeed, in youth populations, past research has demonstrated that tangible interfaces can increase youth engagement and reflection in active learning [40,41], with several learning theories placing emphasis on tangibles as tools to stimulate intellectual development in youths [42-44]. Building on these conclusions, more recent formative research has demonstrated that youth have the ability to conceptualize PA data represented as 3D-printed objects [45]. Moreover, 2 age-specific 3D model representations of youth PA data were developed from formative research [45], which were further validated as a potential tool to increase youth awareness and understanding of PA and the recommended guideline [46]. However, the efficacy of the designed age-specific 3D models in a real-world setting as a tool to enhance youth awareness and understanding of PA is currently unknown.

In accord with Forlizzi and Battarbee [47], understanding how a user’s experiences change over time in connection to a newly designed product is essential for developing the scalability and potential use of the technology in a realistic context. The user’s experience, within the context of technology, is defined by a user’s internal state (perceptions, expectations, motivation, and mood), the characteristics of the product (usability, functionality, and purpose), and the context (organizational or social setting) within which the interactions occur with the technology [48]. More recently, video interview methods have become increasingly popular among researchers to assess a user’s experiences, understanding, and navigation of newly designed technology [36,49,50]. However, these aforementioned video interviews have either been long in duration (eg, 60 min) [36,49] or been implemented with small numbers of individuals (eg, 16-22 participants) [36,50], which may affect the generalizability of findings.

### Objectives

Therefore, the aim of this study was to examine the efficacy of the age-specific 3D-printed models to enhance children and adolescents’ levels of awareness and understanding of and motivation for PA during a 7-week faded intervention, whereby the youths receive personalized 3D-printed models displaying their PALs. It is hypothesized that receiving personalized 3D-printed PA feedback will enhance the youths’ (1) awareness of their MVPA levels compared with the government guideline of 60 min of MVPA; (2) understanding of what constitutes PA and of moderate-and-vigorous-intensity activity; and (3) motivation to be more physically active.

## Methods

### Participants

A total of 2 primary schools and 1 secondary school in South Wales, United Kingdom, were invited to participate in the intervention study. In total, 97 youths participated in the study, of which 39 were primary school children (22 boys; mean age 7.9 [SD 0.3] years) and 58 secondary school adolescents (37 boys; mean age 13.8 [SD 0.3] years). All primary school children were white British, with 96% of secondary school adolescents being white British and the remaining 4% being Asian (2%; n=1) and black British (2%; n=1). All participants returned informed parental or carer consent and child assent before participation. Ethical approval was granted by the University Ethics Committee and conducted in accordance with the Declaration of Helsinki (ref: PG/2014/40).

### Intervention Design

**Figure 1:**
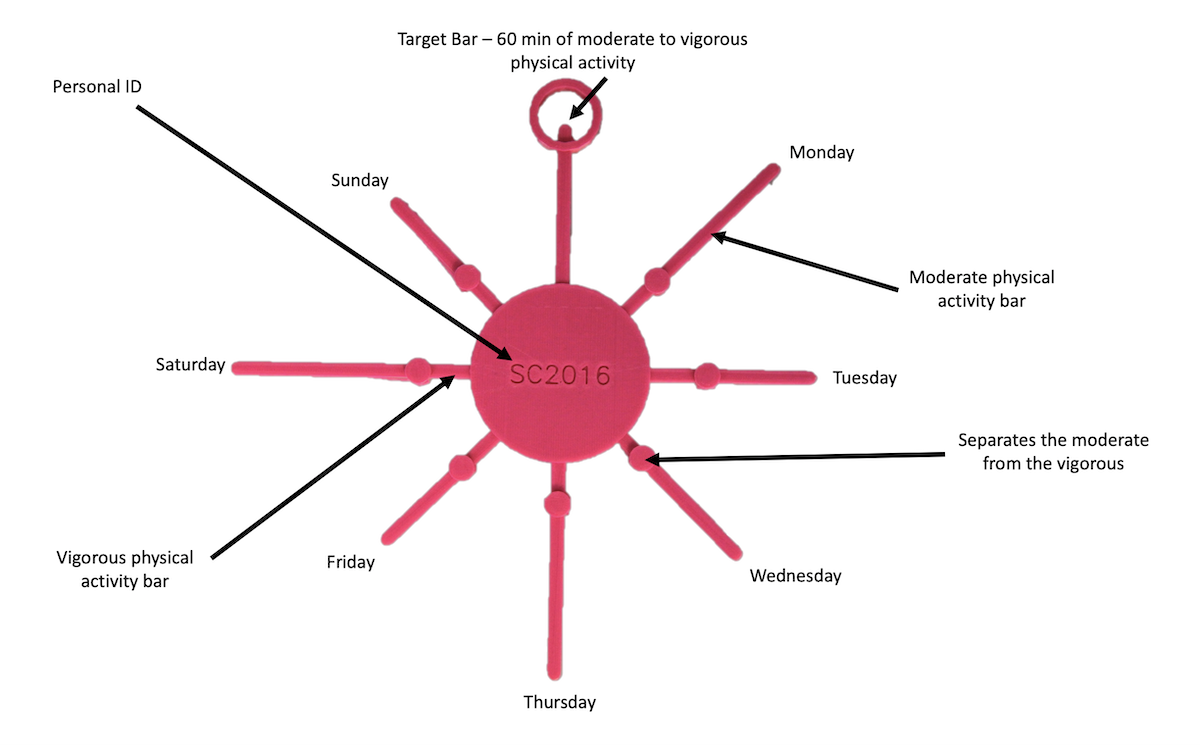
Children’s Sun age-specific three-dimensional model of physical activity instruction manual. 3D: three-dimensional; PA: physical activity.

**Figure 2:**
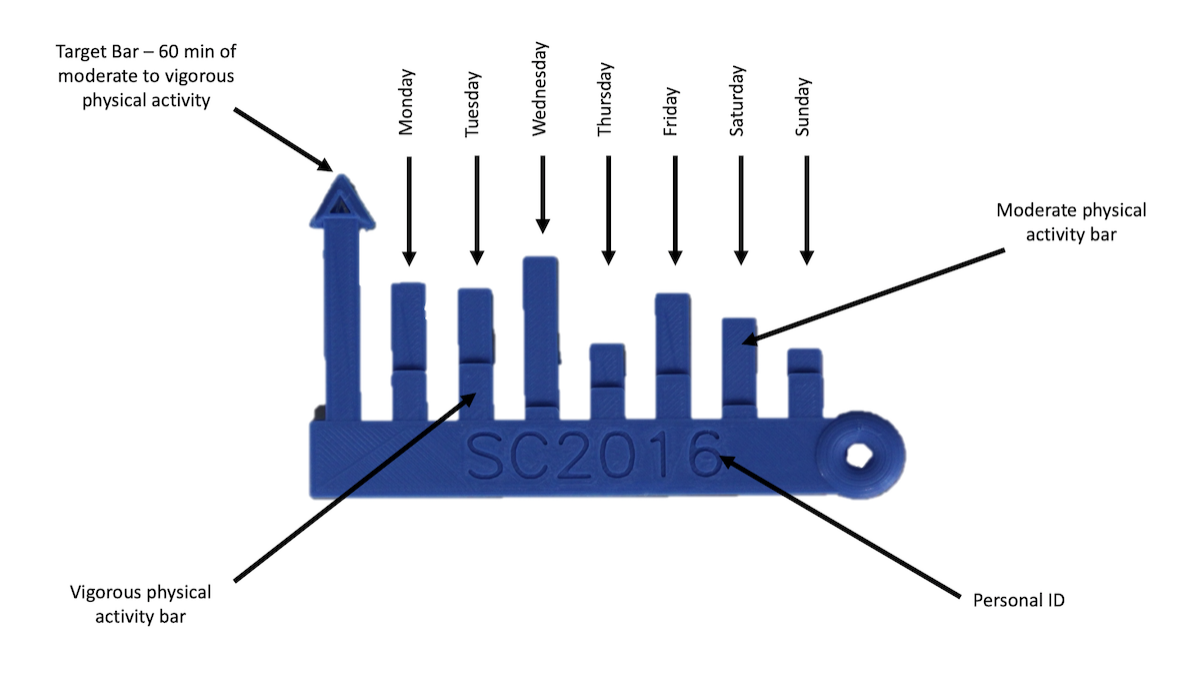
Adolescents’ bar chart age-specific of physical activity instruction manual. PA: physical activity.

The 3D-printing PA intervention was informed by 2 previous user-centered, qualitative approaches that explored the needs, preferences for content and designs, and understanding of 3D-printed models among the youths (ie, children and adolescents) as described in detail elsewhere [45,46]. To encourage lifestyle change, the intervention was theoretically based, in part, on the notion of youths being visual and tactile learners [42,51,52], with an emphasis on the PAPM [22] and Goal Setting Theory [24] as ideologies to enhance awareness of behaviors in relation to set goals through personalized feedback that encompasses a physical incentive. The intervention was implemented for 7 weeks to align with the school term time. The intervention was designed to objectively measure the youths’ weekly PALs and use these data to generate personalized age-specific 3D-printed models to represent the amount of moderate PA and vigorous PA achieved each day across a week as well as display the PA guideline of 60 min of MVPA (Figures 1 and 2). The intervention employed a novel approach that involved participants receiving a total of 4 age-specific 3D-printed models over the course of the 7-week intervention in a faded manner. Specifically, the youths received their 3D models following baseline (model 1=M1), week 1 (model 2=M2), week 3 (model 3=M3), and after week 6 (model 4=M4). The faded approach has been proposed as a method for maximizing the long-term effectiveness of feedback compared with frequent feedback, which only provides short-term benefits [53]. In this regard, the faded method is underpinned by starting with high levels of feedback and then, as the participant begins to master the components of the task, gradually reduce or fade the feedback until the person is performing the task autonomously [54-58]. A key point to this faded design is to increase the sustainability and real-world *implementability* of 3D-printing PA interventions by examining how the 3D models can be integrated into the everyday lives of youths to determine the success of deployment and adoption of the models [59]. Participants received their personal 3D-printed model 1 to 3 days after PA measurement. Immediately following the receipt of each 3D model, all participants completed an individual, semistructured short video interview conducted by the first author either during their physical education class (secondary school) or in an appropriate quiet area within the school environment (primary school) to elicit information on study outcomes [60]. Video interviews are considered a viable method for recording the youths’ experiences with technological designs [50]. All participants received 1 instruction manual (Figures 1 and 2) for their respective age-specific 3D model after completing their first short individual interview to obtain baseline perceptions of primary outcome measures.

### Procedures

#### Anthropometrics

All the participants’ standing stature, body mass, and waist circumference were measured according to the techniques outlined by the International Society for the Advancement of Kinanthropometry [61]. Participants were required to be in minimal clothing (ie, shorts and T-shirt) and barefoot. Body mass was measured to the nearest 0.1 kg using an electronic weighing scale (Seca 876), with stature assessed to the nearest 0.1 cm using a portable stadiometer (Holtain Sitting Height Stadiometer, Holtain Ltd). Body mass index (BMI) and weight status were calculated from stature and body mass measurements as a proxy for adiposity [62]. Based on BMI z-score calculations, age- and sex-specific BMI cut-points from the United Kingdom were applied to categorize participants as underweight, normal weight, or overweight/obese [63]. All anthropometric measurements were conducted within the school by trained research assistants under the supervision of the first author.

#### Measuring and 3D Printing of Physical Activity Data

All participants were asked to wear the wGT3X-BT triaxial accelerometer (ActiGraph LLC) on an elastic belt positioned on their right midaxilla line at the level of the iliac crest for 7 consecutive days to provide an objective estimate of their PALs. Numerous studies have validated the wGT3X-BT triaxial accelerometer as a valid and reliable objective measurement of the quantity and frequency of PA [64-66], with previous research demonstrating that the hip placement is the most precise single location to detect everyday activities [66,67]. All participants were shown a demonstration of the accelerometer hip placement via Sam Graeme Morgan Crossley and provided an information sheet regarding the use and safety of the device at baseline measurement. As far as practically possible, participants wore the same accelerometer (serial number) at each time point to remove *between-unit* variation [68]. Participants were instructed to wear the accelerometer all the time (24 hours per day), except for when engaging in water-based activities (swimming, showering, and bathing) and contact sports. Accelerometers were activated to run from midnight the day participants received the accelerometer until midnight 7 days later and initialized to record raw accelerations at a frequency of 100 Hz. Following the collection of accelerometers, participants’ 7-day PA data were then downloaded and analyzed using Actilife version 6.13.3 (ActiGraph LLC). Given the intervention was designed to provide all participants with raw feedback on MVPA levels, even if the accelerometer was removed (eg, for water-based or contact sport activities), no inclusion criteria were applied to the accelerometry data. Therefore, implications for the youths not wearing the accelerometer on one or more days would result in them receiving a 3D model with no data displayed on that specific day. Each day’s MVPA level was calculated using Evenson child cut-points [69], which are known for providing the closest estimates of moderate-and-vigorous-intensity PALs during the free-living measurement [70]. Participants’ MVPA levels and personal ID code (eg, participant initials and model number) to distinguish participants’ personal age-specific 3D model were then inserted into the age-specific custom-developed 3D model code loaded on OpenJSCAD version 1.8.0 and subsequently 3D-printed using ABSplus filament on the Objet 1000 (Statasys).

To examine participants’ baseline MVPA, data were further analyzed using KineSoft (version 3.3.67; KineSoft), employing 1-second epochs with sustained periods of at least 20 min at zero counts considered as nonwear time [71]. Participants were included in the analysis if they met the minimum daily wear-time criteria of 10 hours for any 3 days [72], which has previously been shown to produce reliable estimates of PA in youth [73]. PA intensities were calculated using cut-points in a study by Evenson et al [69]. Data collection took place during the school term from January to April 2017; therefore, PA data were representative of usual winter or spring free-living activities.

#### Short Individual Video Interviews

Short, individual interviews were chosen as they lend greater control to the interviewer over the interview process relative to the unpredictable nature of focus group interactions [74]. Individual interviews also allow the researcher to locate specific ideologies within particular individuals [75], which is not always possible within focus groups given that the youths may tag onto the views of others without necessarily reflecting on the value or meaning [76]. To reinforce the interpretation of the qualitative data, each individual interview was filmed to capture the youths’ nonverbal and contextual understandings of the 3D model that could be missed in a narrative statement alone [77]. The interviews were semistructured so that the facilitator could ask probing questions around the predefined topics and to keep discussions relevant to the study aims [78]. The 2 interview types (children and adolescents) were conducted using the same research protocol and followed a predefined schedule of questions (Table 1) that sought to address concepts on youth, awareness of the youths' PALs, understanding of intensities and interpretations of the 3D model, and motivational benefits and utility of the 3D models. A total of 369 interviews were digitally voice-recorded (Olympus DM-520 digital voice recorder) and video-recorded (Sony Handycam HDR-PJ540), lasting 4.5 [SD 1.2] and 2.2 [SD 0.6] min, for children and adolescents, respectively. All interviews were transcribed verbatim, resulting in 816 pages (386 and 430 pages for children and adolescents, respectively) of raw transcription data, Arial font size 12, and double spaced.

**Table 1:** Example interview questions.

Topic	Examples
Motivation or awareness of PALs^a^	What do you think of your first 3D^b^ model?
Understanding of PA^c^	What you think physical activity means?
Awareness of PAL or understanding of model	How does your 3D-printed model show your physical activity?
Understanding of intensity	What kind of activities might be vigorous and moderate physical activities?
Motivation or model utility	What will you do with your 3D model now?

^a^PALs: physical activity levels.

^b^3D: three-dimensional.

^c^PA: physical activity.

### Data Analysis

A Shapiro-Wilks test was used to confirm data normality within the anthropometric and PA data. Once normal distributions were confirmed, independent sample *t* tests were used to assess differences between sexes within children and adolescents. All statistical analyses were conducted using IBM SPSS Statistics 22 (SPSS Inc), and statistical differences were accepted at *P≤*.05. Through the process of content analysis, transcripts were approached qualitatively to focus on the context of the youths’ awareness of their PALs and preunderstanding of intensities and the motivational aspects of the 3D models. To quantify patterns within the different time points (ie, receiving model 1-4), it was quantitatively noted as to the number of participants who were associated with specific statements and for the classification of categorical data being accurate (ie, correct interpretations of the 3D model and activity intensities) [79]. To aid and align the accurate classification of 3D model interpretations, interview videos were also assessed to examine participants’ nonverbal interactions with their 3D model by noting gestures (eg, correctly points to the 60-min MVPA guideline bar) within transcripts [77]. All transcripts were thematically analyzed by the first author, first by data immersion, which involved *repeated reading* of the transcripts in an active way, searching and noting of meanings and patterns within the dataset [80]. Following the initial data immersion process, coding was undertaken, using a manual cut and paste technique, which allowed for the data to be organized into groups that were considered pertinent to the research questions. All codes were then sorted into potential themes by collating all relevant coded data extracts to the newly identified theme. The frequency counts and themes with indicative quotes were then represented diagrammatically using a pen profile approach [14,81-83], with the percentages of youth expressing specific themes calculated from frequency counts. The first author discussed the identified themes with the last author to determine the existence of relationships within the data. Themes that did not have enough supportive data or were too diverse were discarded. The second author critically cross-examined the data through reverse triangulation, from the pen profiles back to the transcripts, until all alternative interpretations of the data were exhausted. The pen profiles were then critically reviewed by all other authors, allowing further interpretations of the data until a final consensus was reached.

## Results

### Descriptive Information

Participants’ anthropometric characteristics and PALs are displayed in Table 2. There were no significant sex differences between children, but adolescent boys were significantly taller and heavier than their female counterparts. At baseline, 13% (5/39; boys, 13%, 3/22; girls, 12%, 2/17) of children were overweight or obese with the remaining 87% (34/39; boys, 87%, 19/22; girls, 88%, 15/17) of children being classified as normal weight, with no children being underweight. For adolescents, 22% (13/58; boys, 16%, 6/37; girls, 33%, 7/21) were overweight or obese, and 78% (49/58; boys, 84%, 31/37; girls, 67%, 14/21) were normal weight, with no individuals categorized as underweight. Valid baseline accelerometer data were collected from 68% (66/97) of the consenting participants, with 72% of both children (28/39) and adolescents (42/58) meeting the wear-time criteria. Irrespective of age, there were no significant differences between sexes for baseline MVPA. The provision of baseline MVPA data showed that only 38% (15/39) and 26% (15/58) of children and adolescents, respectively, met the recommended daily MVPA guideline.

**Table 2:** Descriptive and anthropometric characteristics of participants.

Characteristics		Primary			Secondary		
		Boys (n=22)	Girls (n=17)	Both (n=39)	Boys (n=37)	Girls (n=21)	Both (n=58)	
Age (years), mean (SD)		7.9 (0.3)	7.8 (0.35)	7.9 (0.3)	13.8 (0.3)	13.7 (0.3)	13.8 (0.3)	
Stature (m), mean (SD)		1.28 (0.1)	1.25 (0.1)	1.27 (0.1)	1.66 (0.1^a^)	1.63 (0.1)	1.65 (0.1)	
Waist circumference (cm), mean (SD)		58.1 (4.9)	59.6 (5.1)	58.7 (5.0)	73.3 (6.0)	69.2 (6.3)	72.1 (6.4)	
Body mass (kg), mean (SD)		26.1 (3.5)	25.8 (4.0)	26.01 (3.5)	56.05 (10.2^a^)	55.8 (6.8)	55.9 (9.0)	
BMI^b^ (kg/m^2^), mean (SD)		15.9 (2.0)	16.6 (2.4)	16.2 (2.03)	20.2 (2.4)	21.1 (3.0)	20.55 (2.7)	
**Weight status n (%)**							
	Underweight	—^c^	—	—	—	—	—
	Normal weight	19 (87)	15 (88)	34 (87)	31 (84)	14 (67)	49 (78)
	Overweight/obese	3 (13)	2 (12)	5 (13)	6 (16)	7 (33)	13 (22)
**Physical activity levels**							
	Baseline MVPA^d^ (min^e^), mean (SD)	63.3 (11.8)	63.3 (13.4)	63.3 (12.3)	57. 4 (15.4)	50.1 (15.0)	54.6 (15.5)
	MVPA guidelines, n (%)	8 (36)	7 (32)	15 (38)	12 (32)	3 (14)	15 (26)

^a^Significant difference between boys and girls within an age group (*P*<.05).

^b^BMI: body mass index.

^c^Not applicable.

^d^MVPA: moderate-to-vigorous physical activity.

^e^Numbers represent provision of valid days (3 valid days with a minimum 10 hours wear time).

### Primary Outcomes

**Figure 3:**
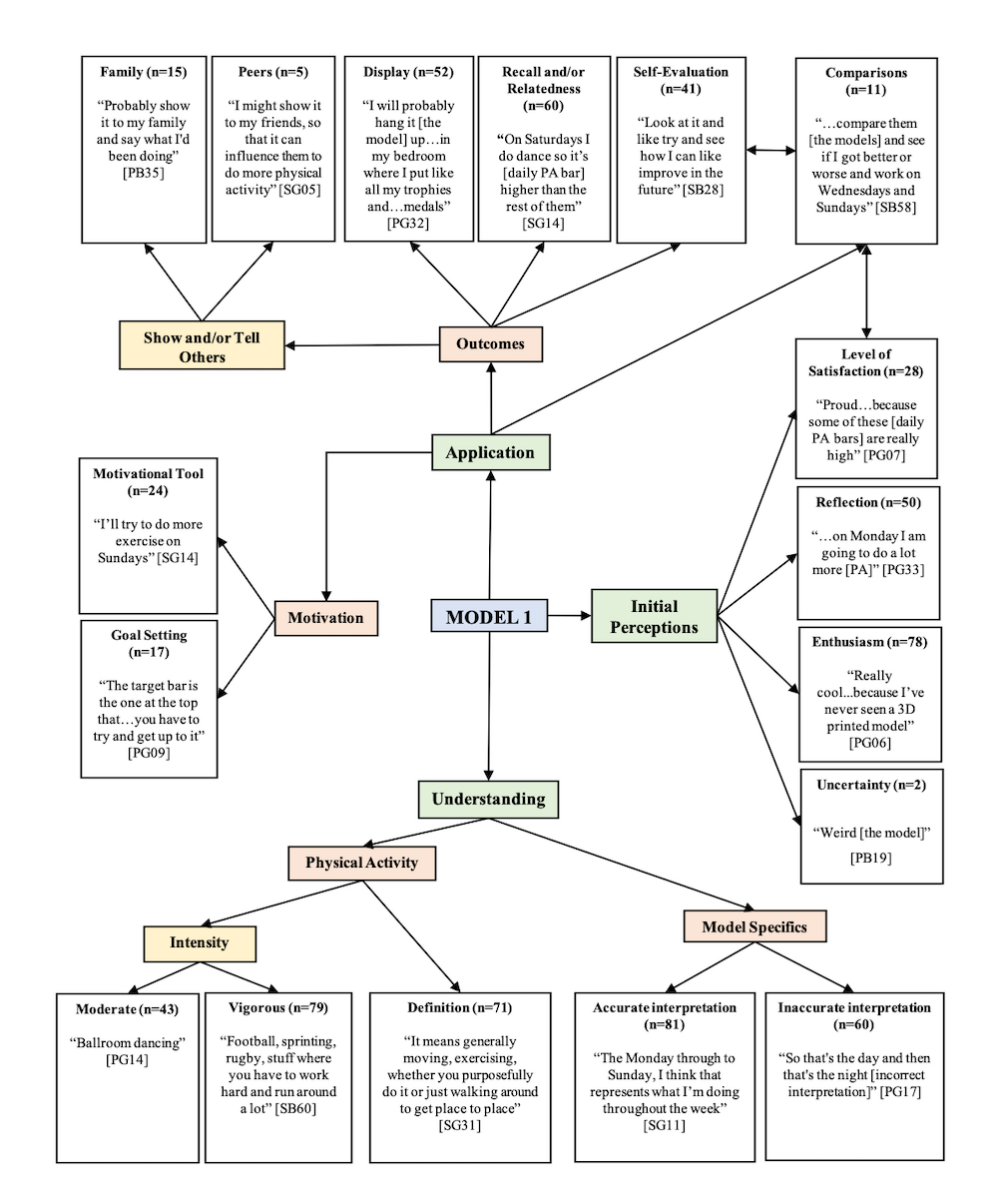
Youths’ pen profile Model 1. 3D: three-dimensional; PA: physical activity.

The first model outcomes for children’s and adolescent’s data are combined and presented in 1 pen profile (Figure 3), as no different themes were found from independent analyses. To avoid duplicating the pen profiles and their identified key themes, Table 3 displays the youths’ frequency of occurrence of key themes for each of the 4 3D models, with children, adolescents, and sex independently split.

Following the first model, the majority of youths (78/97, 80%) expressed a high level of enthusiasm for their 3D model, expressing that it is “really cool...because I’ve never seen a 3D-printed model” (PG06, M1). However, by the final model, only 4% (4/39) of children and no adolescents still expressed similar enthusiasm. Despite this, 28% (28/97) of youths displayed satisfaction on how they were “very proud [of the model]” (PG07, M1) of their first 3D model, with this level of satisfaction increasing from 39% (38/97) to 60% (58/97) and 68% (66/97), by the second, third, and fourth models, respectively. Furthermore, the youths demonstrated increased levels of reflection through the 3D models upon how they “...never thought Saturday was going to be that long” (PB35, M3), from 51% (50/97) to 60% (62/97) and 66% (64/07), for the first, second, and third models, respectively, although by the fourth model, this level of reflection dropped to 58% (56/97).

**Table 3:** The youths’ frequency of occurrence of key themes (n=97). n indicates frequency counts, and superscripted G indicates Girl frequency count.

Themes	Model 1 (n^G^)			Model 2 (n^G^)			Model 3 (n^G^)			Model 4 (n^G^)		
	Children	Adolescents	Total	Children	Adolescents	Total	Children	Adolescents	Total	Children	Adolescents	Total
Enthusiasm	30^15^	48^19^	78	15^4^	4^2^	19	9^3^	0	9	4^1^	0	4
Level of satisfaction	8^3^	20^6^	28	10^2^	28^14^	38	20^10^	38^16^	58	24^9^	42^17^	66
Reflection	13^6^	37^12^	50	24^7^	38^18^	62	20^9^	44^15^	64	14^6^	42^16^	56
Uncertainty	2	0	2	2	0	2	0	0	0	0	0	0
Definition	26^7^	45^18^	71	27^9^	41^14^	68	32^11^	42^17^	74	30^11^	44^15^	74
Moderate intensity	5^2^	38^15^	43	15^5^	34^12^	49	9^6^	36^15^	45	12^2^	34^14^	46
Vigorous intensity	29^10^	50^19^	79	31^10^	47^17^	78	33^12^	49^20^	82	32^13^	47^20^	79
Accurate interpretation	34^14^	47^18^	81	36^14^	46^17^	82	35^14^	51^21^	86	36^14^	49^19^	85
Inaccurate interpretation	33^13^	27^9^	60	19^6^	27^9^	46	21^9^	29^10^	50	18^7^	32^11^	50
Comparisons	1	10^3^	11	20^8^	27^10^	47	20^6^	30^13^	50	14^4^	28^14^	42
Goal setting	10^2^	7^3^	17	21^9^	24^8^	45	31^13^	43^16^	74	31^14^	40^15^	71
Motivational tool	5	19^7^	24	11^2^	19^10^	30	18^7^	22^11^	40	11^2^	23^9^	34
Recall and/or relatedness	18^6^	42^15^	60	19^7^	38^7^	57	28^12^	40^16^	68	30^12^	43^17^	73
Self-evaluation	11^4^	30^11^	41	20^6^	39^15^	59	22^9^	44^18^	66	18^6^	39^17^	57
Display	23^10^	29^10^	52	25^10^	21^5^	46	28^14^	23^7^	51	24^12^	20^8^	44
Family	8^4^	7^4^	15	5^2^	4^4^	9	5^2^	7^6^	12	3	5^4^	8
Peers	3	2^2^	5	1	3^1^	4	1^1^	2^1^	3	1	0	1

Overall, the youths showed little difference in their interpretations of their meaning for PA (M1, 71/97, 73% to M4, 74/97, 76%), stating it is “like doing sports and stuff that includes moving your body” (PB20, M2), with similar outcomes on their interpretations of the intensities of moderate (M1, 43/97, 44% to M4, 46/97, 47%) “like walking” (SB55, M2) and vigorous (M1, 79/97, 81% to M4, 79/97, 81%) “like sprinting so your heart rate is like beating at a fast pace” (SB45, M3). Moreover, across all time points, only 5% (2/39) of children and 17% (10/58) of adolescents were able to relate the guideline bar accurately to “60 minutes of exercise a day” (SG42, M3), with only a small proportion of adolescents (3/58, 5%) able to articulate the guideline of “...at least an hour of hard and moderate activity every day” (SB49, M3). However, the youths demonstrated an accurate ability to interpret the basic components of the 3D models (eg, days and high and low PALs) from the first (81/97, 83%) to the fourth model (85/97, 88%), such as “It [the model] means the days of the week and how much activity you’ve been doing” (PG31, M1), with adolescents being able to correctly distinguish “this one [vigorous bar] is the high-intensity sport activities and this one [moderate bar] is the more moderate sport activities” (SB03, M4). Moreover, the youths were able to correctly interpret and identify with “the target bar...that shows how much exercise you should do in a day, which is one hour” (PB10, M3). As a consequence, the youths increasingly adopted the guideline bar as a goal-setting strategy, from the first (17/97, 18%) to the second (45/97, 46%) and third models (74/97, 76%), with a small drop following the fourth model (71/97, 73%). Specifically, the youths demonstrated this goal setting through how “I haven’t reached the target point [on] Monday” (SG09, M3) and “you have to try and be higher than that arrow [guideline bar] and that would be you reaching your target” (SG35, M4). Conversely, some youths expressed inaccurate interpretations of their 3D models; however, this number dropped with time from the first (60/97, 62%) to the fourth model (50/97, 52%). Of note were the small number of children (10/38, 26%) by the final model who were able to correctly interpret the moderate and vigorous bar representations, with children most commonly mistaking the bar as “the morning [vigorous bar] and that’s the afternoon [moderate bar]” (PB08, M3). For adolescents, only 14% (8/58) demonstrated to incorrectly identify “the lower solid bar [vigorous bar] is walking activity, and the higher bar [moderate bar] is like sprinting activity” (SB52, M4).

**Figure 4:**
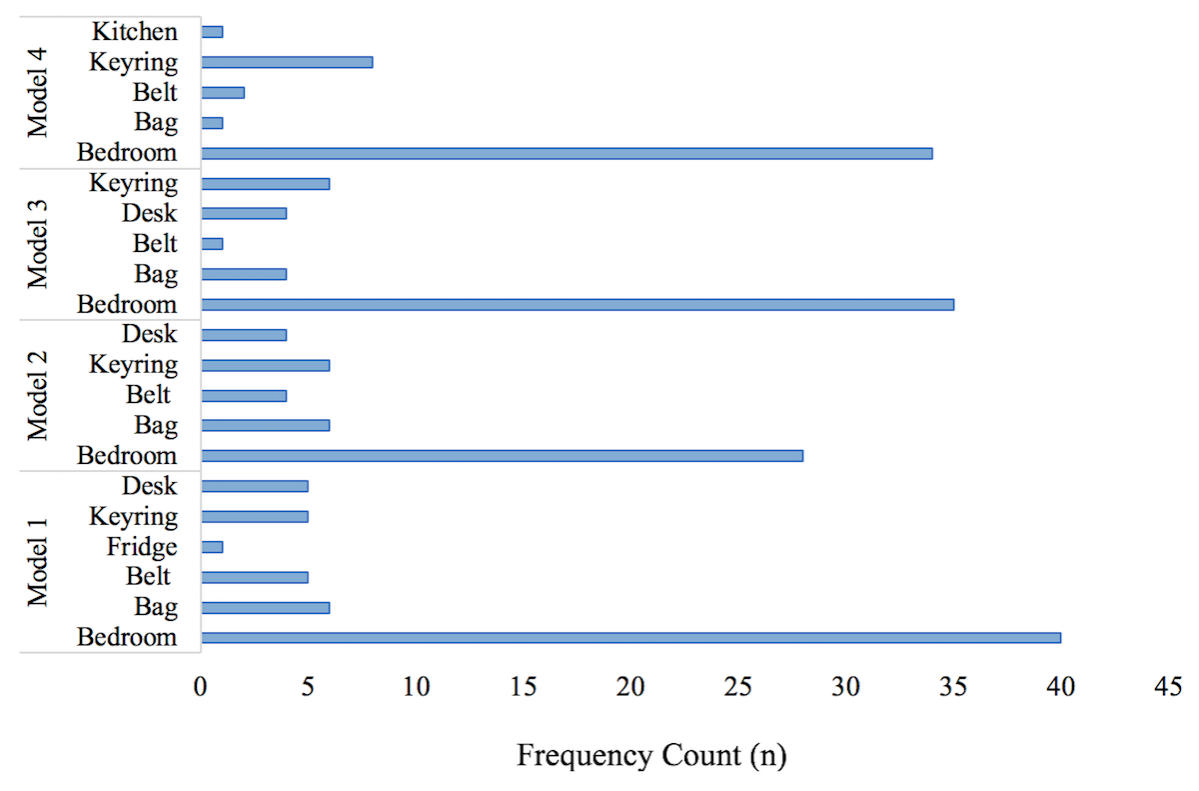
Youths display preferences for three-dimensional models. 3D: three-dimensional.

In terms of the application of the 3D models, 11% (11/97) of the youths expressed that they would “compare the next one [3D model] with it [the current model], and I’ll try to do more exercise on Sundays” (SG14, M1), with this application of the models increasing following the second model (47/97, 48%), with no substantial change for time points thereafter. From the first model, 42% (41/97) of the youths demonstrated self-evaluation of their PALs on how “I need to improve certain days and do more on certain days than others” (SG32, M1), with this self-evaluation increasing from 61% (59/97) to 68% (66/97) for the second and third models, respectively. Interestingly, a higher percentage (18/21, 86%) of adolescent girls appeared to self-evaluate their PALs, expressing they would “see if there’s anything I can change to get a higher activity than what I got” (SG37, M2).

Throughout all time points (M1, 60/97, 62%; M2, 57/97, 59%; M3, 68/97, 70%; M4, 73/97, 75%), there was little change in the youths’ ability to recall and relate their 3D models to their past week of PA, expressing how “on Saturdays I do dance so it’s bar of activity is higher than the rest of them” (SG14, M1). Some youth reported the use of the 3D models as a motivational tool because “it’s [the 3D model] kind of encouraging me to do more activity, so I can get the bar higher [on the 3D model]” (SB19, M2), with this perception increasing from 25% (24/97) to 31% (30/97) to 41% (40/97) for the first, second, and third models, respectively. From all time points, only 5% (5/97) of the youths expressed that they would “show it [the 3D model] to my friends” (PB01, M1), with a larger number of the youths (11/97, 11%), of which were highly representative of adolescent girls and children of both sexes, expressing how they would “probably like show my parents the model” (SG43, M3). Almost half the number of the youths (47/97, 48%) mentioned that they would display their 3D models in their house (Figure 4), with this proportion slightly greater in children, with a preference to “hang the model up in my bedroom” (PB11, M2).

## Discussion

### Principal Findings

The primary aim of this study was to evaluate the effectiveness of 3D models of PA to enhance youth awareness and understanding of and motivation to engage in PA. Taken together, the findings suggest that the 3D model feedback offered a unique strategy to enhance youth awareness of their PALs and associations to the government guideline as well as provide the youths with a motivational tool for goal setting.

In this study, 63% (62/97) of the youths demonstrated that they were able to quickly interpret the basic components of their first 3D model (eg, the different days of activity and their low and high PALs). Indeed, these initial interpretations of the age-specific 3D models are promising given that previous research highlights that being able to quickly interact and interpret a tool, such as 3D model, enables an individual to learn about their behaviors from the start, all of which makes the experience with the tool rewarding and minimizes the potential for abandonment [47]. Following the receipt of their final 3D model, 59% (57/97) of the youths self-evaluated how the 3D models had made them “more aware” (SB58, M2) of their PALs. It could be argued that this raised awareness was a direct result of wearing the accelerometer rather than the 3D model per se; however, this is unlikely, as evidence suggests that accelerometers alone do not develop youth awareness of PA [84]. A more likely reason for this increased awareness may have been the utilization of an objective measure of PA in combination with personalized feedback, which has previously been suggested as an effective combination to raise an individual’s awareness of their PA [85]. Complementary to this understanding, the PAPM [22], from the Stages of Change [23], suggests that an individual is unlikely to proceed to the contemplation stage unless they become aware that their behaviors are inadequate. Base on this notion, this study demonstrated that 68% (27/39) and 78% (45/97) of children and adolescents, respectively, were able to identify that “some days I’m reaching the guideline bar, but some days I need to do more physical activity” (SB51, M4). This ability to apply their respective 3D model guideline bar to their personal PALs is a positive indicator, given that previous research has shown that the youths who are aware of their PALs and the recommended guideline are on average 20 min more active than their unaware counterparts and, consequently, more likely to achieve the 60 min of MVPA [8,86-88]. Therefore, given that awareness of risk behaviors is identified as an independent correlate for behavior change [89], the 3D-printed feedback may not only be important to help the youths categorize themselves into the correct stage of change (ie, precontemplation, contemplation, and preparation=not meeting the guideline, vs action and maintenance=meeting the guideline) but also to help the youths perceive the need to change behavior [90], warranting further investigation.

One important consideration with regard to 3D-printed feedback is that it possesses a higher level of visibility compared with digital feedback (ie, on a mobile phone) within the physical world [91]. In this way, 3D-printed PA data are more publicly visible to peers, teachers, and family members. In contrast to previous perceptions [45], only 5% (5/97) and 11% (11/97) of the youths in this study reported that they compared their models with their peers’ models and showed their family members the models, respectively. Despite this, it could be speculated from previous research that the youths may have more frequently compared their 3D models with those of peers within the playground and classroom environments [92]. Moreover, it is also likely that family members did indeed come into regular contact with the 3D models, given the range of ways that the youths (53/97, 54%) displayed their models in the bedroom, on their school bag, or attached to the house keys. In this regard, it is important to consider how the visibility of the 3D models may have stimulated more social interactions with peers and family and thus influenced the youths’ levels of self-evaluation (58/97, 59%, M4) and reflection (57/97, 58%, M4) of their PALs, rather than the 3D model itself. Indeed, the involvement of peers [93-95] and family [94,96-99] can play a significant role in motivating the youths to be more engaged in PA. On the contrary, sharing and comparing 3D models with peers may induce a competitive environment, which can lead to negative feelings of the self and peer pressure to engage in an activity [100]. Of concern are adolescent girls, who have been shown to be particularly vulnerable at this age to body dissatisfaction, a time when self-awareness, self-consciousness, and preoccupation with self-image all dramatically increase [101]. Indeed, a number of adolescent girls (9/21, 43%) in this study reflected on how being perceived as physically active according to the 3D model was important because “you’ll be more confident because like people won’t judge you” (SG34, M1) and worried about how “if you’re not active you’ll end up having a very, well kind of not nice figure [*body shape*]” (SG14, M2). As a consequence, the youths who display such feelings of pressure and guilt for not achieving enough PA may remove themselves from engaging in peer comparisons (eg, sharing their PALs with others) altogether [100,102] and abandon the 3D model. These issues do question how public displays of PA data could intrude upon an individual’s privacy [103]. In this light, future research should look to monitor more closely how youth, and in particular adolescent girls, personally reflect and evaluate their PALs with regard to body image and the influence of interactions and support from significant others on PALs.

Following receipt of the final 3D model, 72% (71/97) of the youths had seemingly adopted the guideline bar as a goal-setting strategy, expressing how they monitored their goal-related progress through the guideline bar represented on the 3D models. In this way, the 3D models acted as an important moderator for participant goal attainment, which subsequently led to the youths’ self-determined adjustment of PA strategies (eg, starting to play football) and/or effort levels (eg, try harder to do more exercise) [104,105]. As noted within the Goal Setting Theory [24], and addressed in the Social Cognitive Theory [106], setting specific and challenging (yet achievable) goals with feedback on goal attainment is an important step to enhancing an individual’s self-efficacy (ie, their belief to carry out a behavior) and thus, behavior change. Numerous reviews support the effectiveness of goal setting to promote the youths’ PA engagement [107-109], whereas others suggest that feedback alone has a motivating effect, regardless of whether the feedback is tied to a specific goal or not [110-112].

One particular dimension of the Goal Setting Theory [24] that resonates with this study’s findings is the notion that goal attainment can be enhanced by incorporating feedback with rewards (eg, monetary rewards that are linked to goal achievement). Indeed, throughout the intervention, 57% (56/97) of the youths expressed how they would display their 3D model in their bedroom, with some revealing how they placed their models next to their prized “trophies and medals” (PG32, M1). In this way, it could be argued that 3D-printed feedback is received by the youths as a reward of their PA achievements, which is known to heighten an individual’s success toward a goal as opposed to just setting a goal alone [113]. According to Locke and Latham [114], rewards are important to sustain a person’s interest in PA, which may stand true given the success of incentive-based interventions in promoting PA of youth [115-117]. On the contrary, it is important to consider the influence of a reward or incentive on youths’ intrinsic interest to engage in PA as an explicit means to receiving the extrinsic reward (eg, 3D model) and, once removed, whether their behavior reverts back to baseline [118,119]. However, a recent systematic review provides strong evidence that behavioral incentives are an effective means of encouraging PA in youth, suggesting that there is a wide range of incentive designs that are yet to be explored [120]. Perhaps the novelty of 3D printing PA feedback may offer a greater learning value than previous incentive-based designs, as a result of the 3D models being a blend of a reward, feedback, and goal attainment that embodies personalized data and represents the active self [103]. Therefore, this study supports the utilization of tangible feedback as a novel goal-setting strategy for PA of youth through a reward, feedback, and goal attainment, each of which are known to elicit greater self-efficacy [106,113] and youth engagement within interventions [121]. However, for the aforementioned conclusions to be credible, future research should seek to account for youth MVPA levels in response to the tangible representations of PA guidelines (ie, 60 min of MVPA daily) or personal goals as a tool to support youth engagement and understanding of their PA behaviors.

On the basis of previous *learning styles* that support the use of tangibles to inform intellectual development and enable higher mental functions in youth [40,42-44,122], it was originally postulated that the present 3D-printed feedback of PA may enhance the youths’ comprehension of intensities (ie, MVPA) and associations with the government guideline [46]. However, only 5% (2/39) of children and 17% (10/58) of adolescents, across all time points, were able to interpret the guideline bar in terms of the number of minutes (ie, 60 min), whereas no children and 5% (3/58) of adolescents were able to cite “1 hour of physical activity whether it’s moderate or vigorous” (SB60, M4). These findings align with previous research suggesting that particularly children have a lack of ability to define time [123,124] and intensity in the context of PA [12,13,15-17]. Indeed, these findings fuel the present debate to whether *learning styles*, such as youth being *visual and tactile* learners [42], are effective strategies to enhance an individual’s understanding of information [125]. Previous research has demonstrated that changing the learning mode or strategy for a specific population had little improvement on learning outcomes to justify the time and financial costs involved [126-129]. Therefore, the study’s findings question the use of tangibles as an effective means to enhance the youths’ comprehension of the MVPA terms associated with the guidelines. Future research may wish to explore different 3D model designs using inscriptions of the intensities, moderate and vigorous, on the 3D models to aid the youths’ comprehension of terms.

There are a number of inherent challenges associated with 3D printers and their slow development process that should be noted. Specifically, the process of creating the 3D models, following the downloading, analyzing, and mapping of the youths’ PA data onto the 3D models and subsequent 3D printing, involved a considerable amount of time, which consequently delayed the delivery of feedback to the youths. It could be speculated that this delayed timing of the feedback may have negatively affected youth awareness of their PA behaviors, especially given the evidence of the youths’ limited capacity to recall their previous activities [130-132]. Adding to this issue was the number of youths who played contact activities, which involved “taking it [the accelerometer] off because of rugby training” (SB24, M3) and, consequently, “forgetting to put it [the accelerometer] back on again” (SB25, M3). In this regard, the 3D models did not account for PA in the form of water-based activities and contact sports, which are likely to contribute to daily MVPA, and thus will underrepresent the youths’ achievements and awareness of their true PALs and goal attainment (ie, meeting the MVPA guideline bar), all of which could lead to negative feelings of the self [100]. To counteract such problems, future research should look to implement 3D-printed feedback with wrist-worn, fully waterproof accelerometers as they elicit higher wear-time compliance in youth than hip-mounted devices [133], as well as utilizing diary logs to account for contact sport activities [134]. Nevertheless, it is important to acknowledge that efforts are currently being made to make 3D printers faster, more accurate, and cheaper [135], with the potential for future research to involve youth more in the 3D printing process. Indeed, the number of schools owning a 3D printer is on the rise [136], which makes 3D-printing interventions similar to this study more feasible and cost-effective. In this light, it may be useful to compare 3D-printed feedback with other approaches, such as digital mobile phone feedback [137,138], light-emitting diode feedback technology [139,140], 3D-printed edibles [141], or shape-changing artifacts [142] to determine which methods of feedback can elicit the best intervention effects, user experience, and cost-effectiveness.

According to Forlizzi and Battarbee [47], new research methods are required to better articulate the relationship between what *we feel* and what *we do* in connection to the utilization of technology. This study builds on this by illustrating a short video interview approach to eliciting how youth experienced the 3D-printed models internally, functionally, and socially, all of which is essential for the development and future utilization of the designed 3D models [48]. The short video interviews generated a large set of descriptive data that could be generalized to the study population or used to account for an individual’s personal progress and experiences with the 3D models, which aligns with the current trend toward *personalization* in health care [143] and the *quantified-self* movement [144]. However, one possible limitation to this aforementioned approach could be the direct influence of the ongoing short video interviews on the youths’ experiences with the 3D models, given that previous research suggests that face-to-face support can create a more meaningful experience by reinforcing effort and goals [145,146]. In this regard, it could be argued that the ongoing face-to-face short video interviews may have potentially influenced youth awareness and motivation for PA, rather than the 3D models per se. Indeed, there are a number of practical ways a researcher or health professional could be deployed to support such a feedback intervention; however, to make technology-based behavior change strategies more pragmatic and cost-effective, it would be useful to understand the efficacy of support through continuous interviews [147]. Therefore, future research should look to break down 3D-printed feedback conditions to include and exclude face-to-face interviews to fully understand the impact of the tangible feedback and the influence of regular interviews on outcome variables [148]. That said, this study supports the use of short video interviews as a practical method for assessing the youths’ experiences, understanding of, and interactions with the newly designed technology.

### Limitations

There are, however, some additional limitations to consider to the aforementioned, such as the localized area of data collection in South Wales, which may underrepresent the ideologies of youth from other important socioeconomic groups and ethnic minorities in the United Kingdom or at a global level. Given the paucity of research on 3D-printed feedback, further research is required that considers the influence of age and sex, specifically, which may be hypothesized to influence initial engagement with the models. Indeed, the lack of a control group within this study questions whether the changes observed can be attributed to the impact of the 3D models per se to enhance youth awareness, goal setting, and motivation, and therefore, findings should be considered with caution and act as a stimulus for future investigation. Finally, this study was only a 7-week intervention with no long-term follow-up; therefore, it is unknown to what extent the novelty effect of the 3D models may diminish with time, as previously observed in the youths with wearable activity trackers [92]; therefore, a long-term study is warranted.

### Conclusions

In conclusion, this study demonstrated that the age-specific 3D models heightened youth awareness of their PALs and enabled them to easily compare their personal PALs with the recommended guideline of 60 min of MVPA. Moreover, the youths expressed how they displayed their 3D models in their environments, within their bedrooms or next to prized possessions, and utilized the model as a goal-setting strategy to do more PA. Therefore, the nature of the age-specific 3D models being a combination of feedback, reward, and goal attainment that embodies personalized data may offer a unique strategy for the promotion of PA and associations to the recommended government guideline.

## Abbreviations

3D: three-dimensional
BMI: body mass index
M1: model 1
M2: model 2
M3: model 3
M4: model 4
MVPA: moderate-to-vigorous physical activity
PA: physical activity
PALs: physical activity levels
PAPM: Precaution Adoption Process Model
